# Treatment outcomes of severe acute malnutrition and predictors of recovery in under-five children treated within outpatient therapeutic programs in Ethiopia: a systematic review and meta-analysis

**DOI:** 10.1186/s12887-020-02188-5

**Published:** 2020-07-07

**Authors:** Zebenay Workneh Bitew, Ayinalem Alemu, Teshager Worku

**Affiliations:** 1 Department of Pediatric Nursing, St. Paul’s Hospital Millennium Medical College, School of Nursing, Addis Ababa, Ethiopia; 2 Ethipian Public Health Institute, Addis Ababa, Ethiopia; 3 College of Health and Medical Sciences, School of Nursing and Midwifery, Haramaya University, Harar, Ethiopia

**Keywords:** Survival, Severe malnutrition; cure rate, Determinants, Outcomes; regions of Ethiopia

## Abstract

**Background:**

Severe acute malnutrition affects around 17 million under-five children in the world, of which the highest burden is accounted by Sub-Saharan Africa where Ethiopia is found. Though there are few individual, inconsistent and inconclusive studies, there is no nationally representative study on treatment outcomes of SAM in outpatient therapeutic feeding programs of Ethiopia. This study aimed at estimating the pooled treatment outcomes and predictors of recovery rate among under- five children with SAM in Ethiopia.

**Methods:**

Electronic databases (PubMed, Medline (EBSCOhost), EMBASE (Elsevier), CINAHL (EBSCOhost), web of science, Scopus, Science Direct and Food Science and Technology Abstracts (FSTA)), and grey literature sources (Google scholar, Mednar, World Cat and google) were used to retrieve articles. The random effect model was used to estimate the pooled treatment outcomes. Hazard ratios were used to determine the predictors of recovery rate. Cochran’s Q, I^2^, and univariate Meta regression were done for heterogeneity. Begg’s & Egger’s tests were used for publication bias.

**Results:**

Nineteen articles with a total number of 23,395 under-five children with SAM were used for this meta-analysis. The pooled recovery, death, defaulter and non-recovery rates were 70% (95% CI: 64, 76), 2% (95% CI: 1, 2), 10% (95%CI: 7, 12), 15% (95% CI: 10, 20), respectively. Diarrhea (HR = 0.8, 95% CI: 0.75, 0.94), no edema (HR = 0.41, 95% CI: 0.33, 0.50) and amoxicillin (HR = 1.81, 95% CI: 1.18, 2.44) were independent predictors of recovery rate of children with SAM in Ethiopia. Publication year was found to be the potential source of heterogeneity between included studies.

**Conclusion:**

The treatment outcomes of children with SAM from outpatient therapeutic feeding programs of Ethiopia are lower than the sphere guidelines, WHO and national recommendations. Diarrhea and no edema antagonized the recovery rate of children, while amoxicillin enhanced the recovery rate of children from SAM. Community health workers need to be trained. Especial attention should be given while treating children with diarrhea and severe wasting. Community mobilization is also recommended to improve community awareness about the therapeutic foods.

## Background

Severe acute malnutrition is defined as very low weight for height/ length (<− 3 z score of the median world health organization growth standards or presence of bilateral edema or Mid Upper Arm Circumference < 115 mm for a child ≥6 months age [1]. The magnitude of SAM remains high with an estimated 17 million under-five children were victims of SAM in 2016, of which the majority were from Africa and Asia where Ethiopia is found [2, 3]. Of these, around 16.6 million children were affected by severe wasting [4]. The unwanted treatment outcomes of SAM also remain high throughout the globe which escalates the risk of death by 12 times as compared to well-nourished children [5]. Despite global progress in the number of children treated for SAM (from 1.1 million in 2009 to 4.4 million in 2017), only one in four children receives treatment [4].

Children with SAM get treatment either in the inpatient units or in the outpatient therapeutic feeding programs. Outpatient therapeutic feeding program (OTP) is part community-based management of acute malnutrition (CMAM) for children with uncomplicated severe acute malnutrition (SAM) [6]. The program services include diagnosis and provision of ready-to use therapeutic foods (RUTF) every week for 2 months; supplementation of medications like amoxicillin, folic acid, vitamin-A, measles vaccine and deworming [7].

Prior to the implementation OTP in Ethiopia, children with SAM were treated in the inpatient units with its many limitations [8, 9]. Limited coverage and impact, costliness, cross infections, and high rate of mortality rate were some of the challenges contributing to inefficacy of inpatient management of SAM [10, 11]. Due to those limitations, OTP was endorsed as a health care system since 2005 [12] to treat children with uncomplicated SAM after the pilot that was tested since 2000 [13]. This is because early detection and OTP management is the cornerstone in the modern management of SAM that can limit the number of children in need of inpatient care [14]. Community based management can also help for early detection of children with SAM which could enhance early recovery [15]. Besides, treating SAM children in OTP centers could minimize the costs spent in inpatient based programs [16, 17].

In spite of the fact that OTP has been implemented in Ethiopia from the inception of the program, the recovery rate of under-five children with SAM still remained unacceptably high. This is substantiated by the original studies conducted in different regions of the country with the recovery rates ranging from 32.7% [18] to 92.7% [19]. The recovery rates in most of the original studies [12, 18, 20–28] were below the sphere standard [29]. The death rates also remain significantly high in some OTP centers of the country reaching as high as 14 % [25]. Moreover, a remarkable variation is seen in the defaulting rates and non-recovery rates. The defaulting rate ranged from 1.67% [23] to 25.2% [12] and the non-recovery rate ranged from to 2 % [20] to 61.13% [18]. These inconsistent and inconclusive findings implied as there are unfinished tasks in the management process of SAM in Ethiopia. These inconsistencies could be attributed by multiple challenges during the implementation process of OTP. The common challenges that made the country not on the course of meeting the goals include; food sharing, trading of RUTFs as commodity, high cost of standard RUTFs, stigma associated with RUTFs use, lack of antibiotics, inappropriate exit from the program, and disliking the taste of RUTFs [7, 30]. In-addition, the determinants of treatment outcomes, particularly the predictors of recovery rate are not addressed comprehensively. Few studies [21, 24–26, 31–35] revealed that edema, diarrhea, deworming, giving antibiotics, vitamin A supplementation, and distance from OTP centers and age were predictors of recovery of under-five children with SAM in OTPs of Ethiopia.

Therefore, the main purpose of this systematic review and meta-analysis is determining treatment outcomes and predictors of recovery rate among under-five children with SAM in the outpatient therapeutic feeding programs in Ethiopia. The findings could help policy makers, stakeholders, and community health workers for the appropriate management of SAM at the OTPs.

## Methods

### Searching strategies

In this systematic review and meta-analysis, the preferred reporting items for systematic review and meta-analysis (PRISMA) [36] was followed to write the whole document. All possible studies were retrieved comprehensively from the reputable databases (PubMed, Medline (EBSCOhost), EMBASE (Elsevier), CINAHL (EBSCOhost), web of science, Scopus, Science Direct and Food Science and Technology Abstracts (FSTA)) and grey literature sources (Google scholar, Mednar, World Cat and google). The reference lists of included studies were also checked and searched accordingly. Two author (ZWB & TW) searched studies independently using the key terms: (a) population (infants, toddlers, preschoolers, under-five children); (b) exposure (severe acute malnutrition, SAM, severe malnutrition, protein energy malnutrition, PEM, uncomplicated severe acute malnutrition) (c) outcome (recovery, survival, cure rate, death, non-recovery, non-responder, transfers); (d) study design (cohort, cross-sectional, prevalence, epidemiology, observational); (e) study setting (outpatient treatment program, OTP, community based management of acute malnutrition, CMAM, health posts, health centers) and (f) location (Ethiopia, regions of Ethiopia, parts of Ethiopia). The Boolean search operators such as “OR”, “AND”, “AND/OR” were used during the searching process. The appropriateness of key terms was verified before the actual search was conducted. Literature searches were limited to, articles conducted in the English language. The EndNote X8 reference manager was used to manage the literatures. In this study, studies conducted from 2007 to January 20, 2020 were included.

### Eligibility criteria

#### Inclusion criteria

The two investigators (ZWB & AA) independently assessed the contents of each of the included studies and articles that met the following criteria were included in the final analysis.

#### Population

Studies, which were done among under-five children, were included.

#### Study setting

The studies conducted in areas where OTPs are implemented (health posts, health centers) were considered.

#### Study area

Studies conducted in Ethiopia were included.

#### Study design

Original articles which were conducted both in cross-sectional and cohort study designs measuring treatment outcomes and associated factors were considered.

#### Language

Studies conducted in the English language were considered.

#### Publication condition

Studies fulfilling the predefined criteria (published or unpublished studies) were included.

#### Exclusion criteria

Two authors (ZWB &AA) did data extraction blindly and independently after reviewing the abstracts and full texts of included studies. In addition, methodological qualities of studies were assessed by three authors (ZWB, AA, & TW) independently. We excluded studies that were difficult to access the full text after failing to communicate the corresponding authors.

### Data abstraction and critical appraisal of the studies

Structured and pre-tested data extraction checklist was used to extract the data by two authors (ZWB & AA). The terms included in the extraction checklist were; the name the first author & publication year, study region, study design, study period, study setting, age of study subjects, sample size, treatment outcomes (recovery, death, defaulting, non-recovery, unknown), median recovery days and predictors of recovery. The third author (TW) actively involved in resolving disagreements between two authors. The qualities of the studies were assessed using the Joana Briggs Institute checklists of cross-sectional and cohort studies [37]. Critical appraisal was done by two authors (ZWB & AA), independently and blindly. The tools have Yes/No questions and 1 was given for Yes and 0 for others. The scores were summed up and changed to percentages. Studies with > 50% quality score were included in the meta-analysis (See Additional file 3). The mean scores of the two reviewers were used for final decision of inclusion of the studies in this systematic review and meta-analysis. During critical appraisal, the third author (TW) played a crucial role in solving the incongruities between two authors. The asymmetry of the funnel plot and/or statistical significance of Egger’s regression test (*p* < 0.05) [38] were considered as indicators of publication bias.

### Operationalization of the outcomes

The primary outcome of this study is the recovery rate of under-five children from severe acute malnutrition who were treated from OTP centers of Ethiopia. It was computed by dividing the number of children recovered to the total sample and multiplying it by 100. The second outcome was the predictors of recovery using the hazard ratios from the included studies. The other outcomes were death rate, defaulter rate, non-recovery rate and all were calculated in the same approach recovery rate was calculated. The binomial distribution formula was used to compute the standard errors for each original study. In the current study, children who didn’t respond to the therapeutic foods, those who were referred to the inpatient units due to medical complication and those transferred out before the discharge date were considered as non-recovered cases.

### Data analysis and assessment of certainty in the findings

The data were extracted using an extraction checklist prepared using Microsoft excel 2016 (Table 1). Data were imported into STATA Version 15 (STATA Corporation, College Station Texas) software for analysis of the pooled estimates of recovery rates, death rates, defaulter rates, non-recovery rates and predictors of recovery rate of under-five children with SAM. Forest plot and summery tables were used to report meta-analyses results. The pooled estimates of outcomes and predictors were analyzed with 95% CI. Heterogeneity among studies was explored by using forest plot and I^2^ test and Cochrane Q statistics [39]. The I^2^ values of 25, 50 and 75% were interpreted as low, medium and high heterogeneity, respectively. In this study, the presence of heterogeneity was declared and justified when was I^2^ ≥ 50% and a *P value* of < 0.05. The statistical tests pinpointed that there was heterogeneity [40] among the studies (I^2^ = 98.7%, *P* = 0.000). Random and fixed effect models were used interchangeably in the analyses. Since there was no a significant difference were observed, a random effects model was used to estimate the Der Simonian and Laird’s pooled effect size of recovery rate [41, 42].

**Table 1:** Summary of 19 included studies on treatment outcomes of SAM among under-five children admitted to outpatient therapeutic feeding programs in Ethiopia

Author, Publication year	Study region	Study design	Study period	Study setting	Age (months)	Sample size	Median recovery time in weeks (IQR)	Recovery rate N (%)	Death rate N (%)	Defaulter rate N (%)	Non-recovery N (%)	Quality Scores^a^
Degebasa, 2017 [1]	Tigray	Cohort	2011–2015	HC	6–59	2009	–	1406 (70)	1 (0.06)	74 (3.66)	40 (1.99)	7
Mamo, 2019 [2]	Amhara	Cohort	2017	HC	6–59	389	5.5 (3.5,7.5)	254 (65.3)	–	17 (4.37)	41 (10.54)	10
Boltena, 2008 [3]	SNNP	Cross sectional	2008	HC	< 59	355	–	329 (92.7)	11 (3)	7 (2)	8 (2.3)	6
Kabalo, 2017 [4]	SNNP	Cross sectional	2014	HP	6–59	776	–	504 (64.9)	9 (1.2)	17 (2.2)	246 (31.7)	8
Yebyo, 2013 [5]	Tigray	Cohort	2008–2012	HC & HP	6–59	628	–	388 (61.78)	87 (13.85)	19 (3.02)	56 (8.91)	10
Kabalo,2018 [6]	SNNP	Cohort	2014–2015	HP	0–59	582	–	396 (68)	6 (1.57)	10 (1.72)	170 (29.2)	9
Kabalo,2016 [7]	SNNP	Cross sectional	2015	HP	< 59	600	–	396 (66)	4 (0.7)	10 (1.7)	–	8
Shanka, 2015 [8]	SNNP	Cohort	2011–2013	HC & HP	< 59	711	7.14 (5.28,8.14)	522 (67.7)	13 (1.8)	175 (24.6)	13 (1.83)	10
Atnafe, 2019 [9]	Dre Dawa	Cohort	2013–2016	HC & HP	6–59	713	8.7 (5,14)	569 (79.8)	4 (0.6)	80 (11.2)	42 (5.9)	10
Mengesha,2016 [10]	SNNP	Cohort	2008–2009	HP	6–59	348	6	274 (78.7)	–	–	74 (21.3)	9
Teshome,2019 [11]	SNNP	Cohort	2015	HP	6–59	216	5 (4.67,5.33)	172 (79.6)	–	8 (3.7)	36 (16.7)	9
Liben, 2019 [12]	Afar	Cohort	2017	HC & HP	6–59	286	7 (2.7, 8.1)	238 (83.2)	8 (2.8)	18 (6.3)	22 (7.7)	10
Tadesse, 2018 [13]	SNNP	Cohort	2011	HP	6–59	759	–	248 (32.7)	17 (2.2)	18 (2.4)	464 (61.1)	6
Beletew, 2019 [14]	Amhara	Cohort	2016–2019	HP	0–59	600	–	390 (65)	12 (2)	96 (16)	102 (17)	8
Yorra, 2016 [15]	SNNP	Cohort	2013–2015	HC & HP	6–59	602	–	414 (68.8)	8 (1.3)	145 (24.1)	21 (3.5)	7
Massa, 2016 [16]	Tigray	Cohort	2012	HC & HP	6–59	332	7 (4,8)	255 (76.8)	2 (0.6)	58 (17.5)	17 (5.1)	10
Mokgatle,2015 [17]	Oromia	Cross sectional	2010	HP	6–59	163	–	114 (69.9)	–	36 (22.1)	–	6
Teferi,2009 [18]	SNNP	Cohort	2003–2005	HC & HP	0–59	12,316	–	9871 (80)	217 (2.5)	–	–	8
Belachew, 2007 [19]	AA, SNNP & Oromia	Cross sectional	2006	HC	< 59	1010	–	554 (55)	4 (0.4)	255 (25.2)	197 (19.5)	6

*AA* Addis Ababa, *SNNP* Southern Nations,Nationalities and Peoples, *HC* Health Center, *HP* Health Posts; ^a^The quality scores for cohort studies were computed out of 11 indicators while for cross sectional studies from 8 indicators; *IQR* interquartile range

To identify the possible sources of heterogeneity, meta-regression analysis was done using the sample size and publication year as the possible source of variability. However, sample size was found to be statistically insignificant (*P* = 0.064) and publication year was found to be the possible source of variation (*P* < 0.001) (Table 2). Funnel plot was drawn using recovery rate and standard error of recovery rate it revealed as there is a publication bias (Fig. 3). The possible source of publication bias was also objectively examined using Egger’s weighted correlation and Begg’s regression tests [43]. The result showed that as there is publication bias (*P* = 0.036) in the Egger test and Begg’s test was found to be insignificant (*P* = 0.944). Hence, the pooled estimate of recovery rate was determined using Duval and Tweedie’s Trim and Fill analysis in the Random-effects model. In addition, subgroup analysis was done using the study region and study year. This is done to minimize the random variations between the point estimates of the included studies.

**Table 2:** Factors associated with heterogeneity of recovery rate of children with SAM in Ethiopia (univariate meta-regression)

Variables	Coefficient	*P*-value
Publication Year	2015.4	< 0.001
Sample Size	1231	0.064

## Results

### Selection of eligible studies

**Fig. 1:**
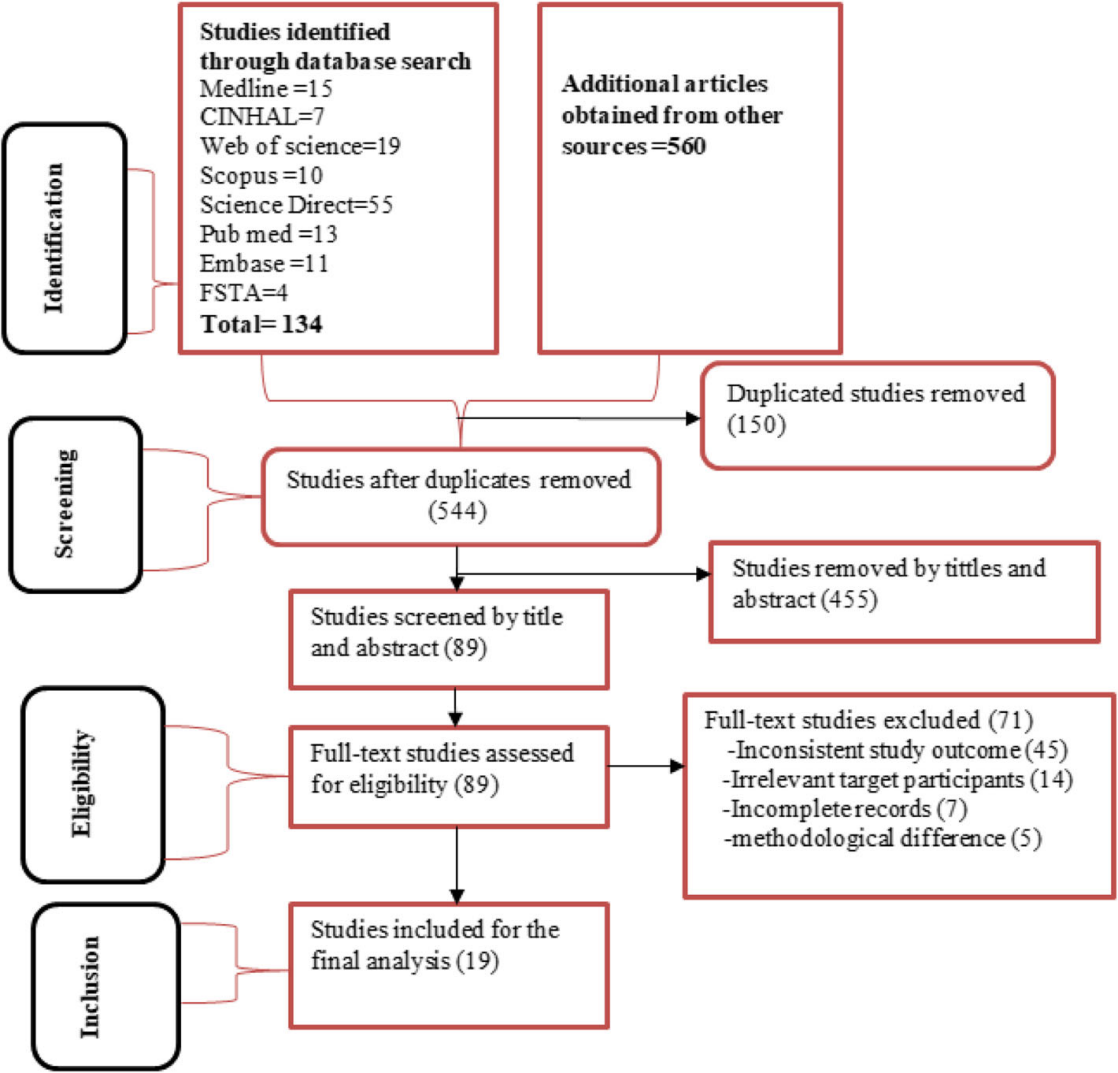
The PRSIMA flow chart showing the selection process of studies

In the first search, 694 studies were found from electronic databases and grey literature sources. Of the total studies, 150 were duplicated files and 455 studies were removed after screening based on titles and abstracts. The full texts of 89 articles were reviewed. Finally, 19 articles [12, 18–28, 31–35, 44, 45] were included in the final analysis of this systematic review and meta-analysis (Fig. 1).

### Characteristics of the original studies

The details of all the included studies are clearly summarized in Table 1. Cohort [18, 20, 21, 24–27, 31–35, 44, 45] and cross-sectional [12, 19, 22, 23, 28] studies were included in this study. Coming to the regional distribution of studies, most of the studies were done in Southern Nations, Nationalities and Peoples of Ethiopia (SNNP) [18, 19, 22–24, 26, 32, 33, 44, 45]. While, three were form Tigray region [20, 25, 35] and two of the studies were conducted in Amhara region [21, 27]. The others were done DireDawa administration [31], Afar region [34] and Oromia region [28]. One study was conducted from patient records in Addis Ababa, SNNP and Oromia [12]. The studies were done with review of documents form health centers and health posts and the sample sizes ranged from 163 in Oromia region [28] to 12,316 in SNNP [44]. The maximum recovery rate (92.7%) was recorded in SNNP and the minimum one (55%) was from the study done in Oromia region, Addis Ababa and SNNP [12]. In this systematic review and meta-analysis, a total of 23,395 under-five children with SAM who were treated in OTPs of different regions of Ethiopia were included. The included studies were conducted from 2007 to 2019. Regarding the quality scores of studies, eleven of them were classified under high quality, whereas, seven and one of them were classified under medium and low qualities, respectively. Moreover, the median recovery times were explored and only seven studies [21, 26, 31–35] reported this. The median recovery time ranged from 5 weeks (interquartile range: 4.67, 5.33) [33] to 8.7 weeks (interquartile range: 5, 14) [31].

### Treatment outcomes of children with SAM in Ethiopia

**Fig. 2:**
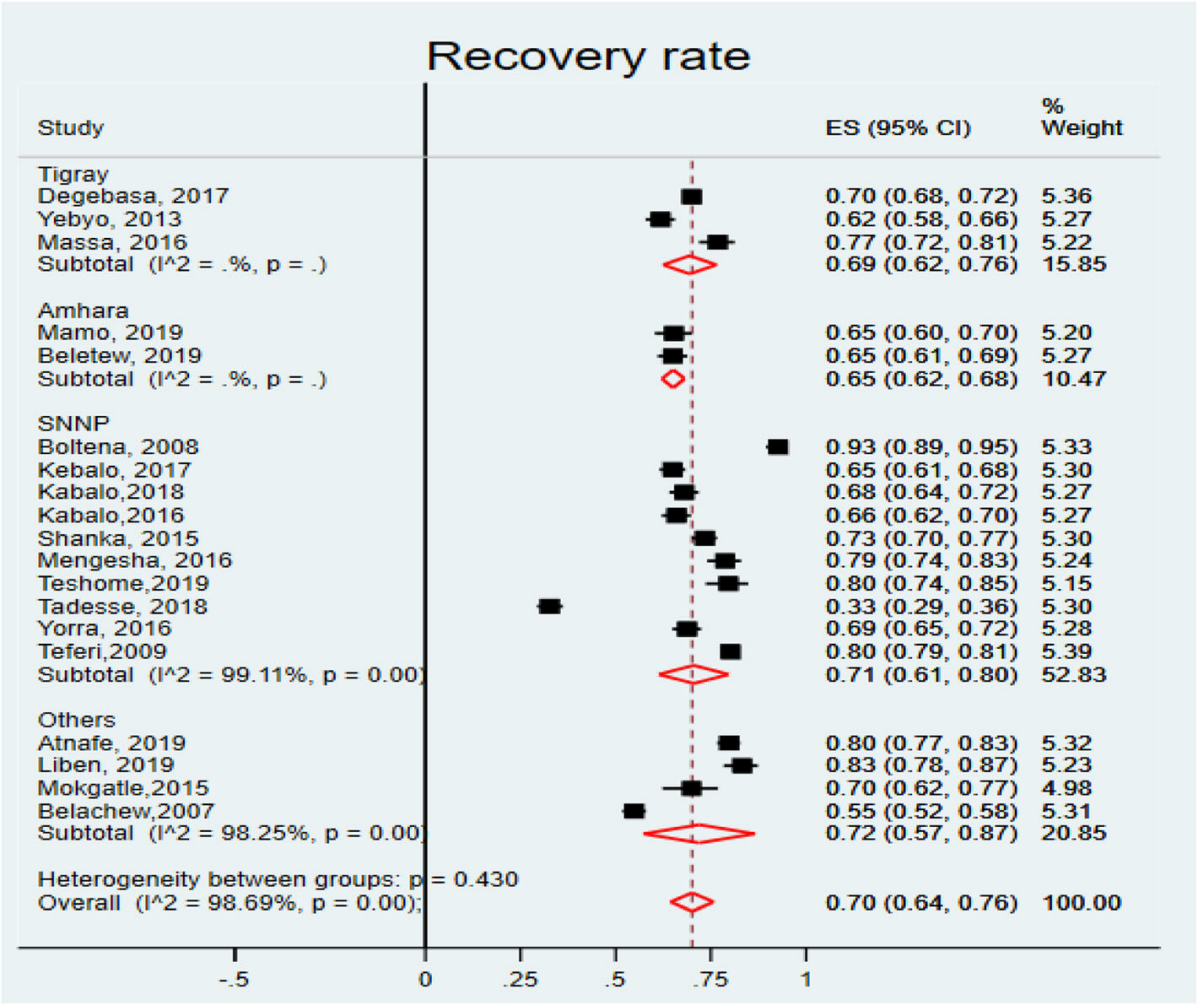
Forest plot showing the recovery rate of children with SAM treated in OTPs of Ethiopia, 2020

**Fig. 3:**
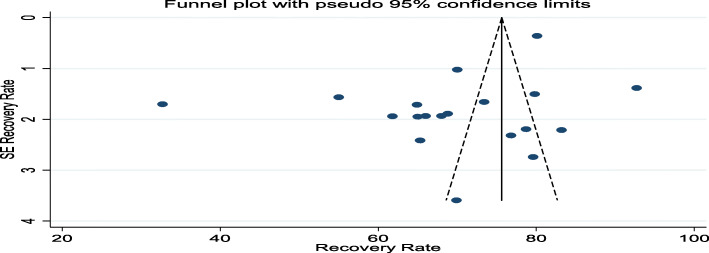
Funnel plot showing the possible source’s bias of recovery rate of children from SAM in OTPs of Ethiopia, 2020

A total of 19 studies [12, 18–28, 31–35, 44, 45] were used to compute the pooled estimate of recovery rate of under-five children with SAM who were treated in the OTPs of Ethiopia. The recovery rate was found to be 70% (95% CI: 64, 76, I^2^ = 98.69% & *P* = 0.000) (Fig. 2). The I^2^ statistic shows significant heterogeneity among studies. Due to this, the possible sources were checked using univariate meta-regression analysis by using publication year and sample size (Table 2). Sample size was found to be insignificantly associated (*P* = 0.064) and publication year was found to be the possible source of variation (*P* < 0.001). Publication bias was checked using funnel plots which showed the possibility of bias (Fig. 3). The presence of publication bias was confirmed by objective measures using Begg’s and Egger’s test. The Egger’s test revealed significant publication bias (*P* = 0.036), but Begg’s test was found to be insignificant (*P* = 0.944). Therefore, Trim and Fill analysis was done to adjust the final pooled recovery rate of children with SAM who were treated in OTPs of Ethiopia.

**Fig. 4:**
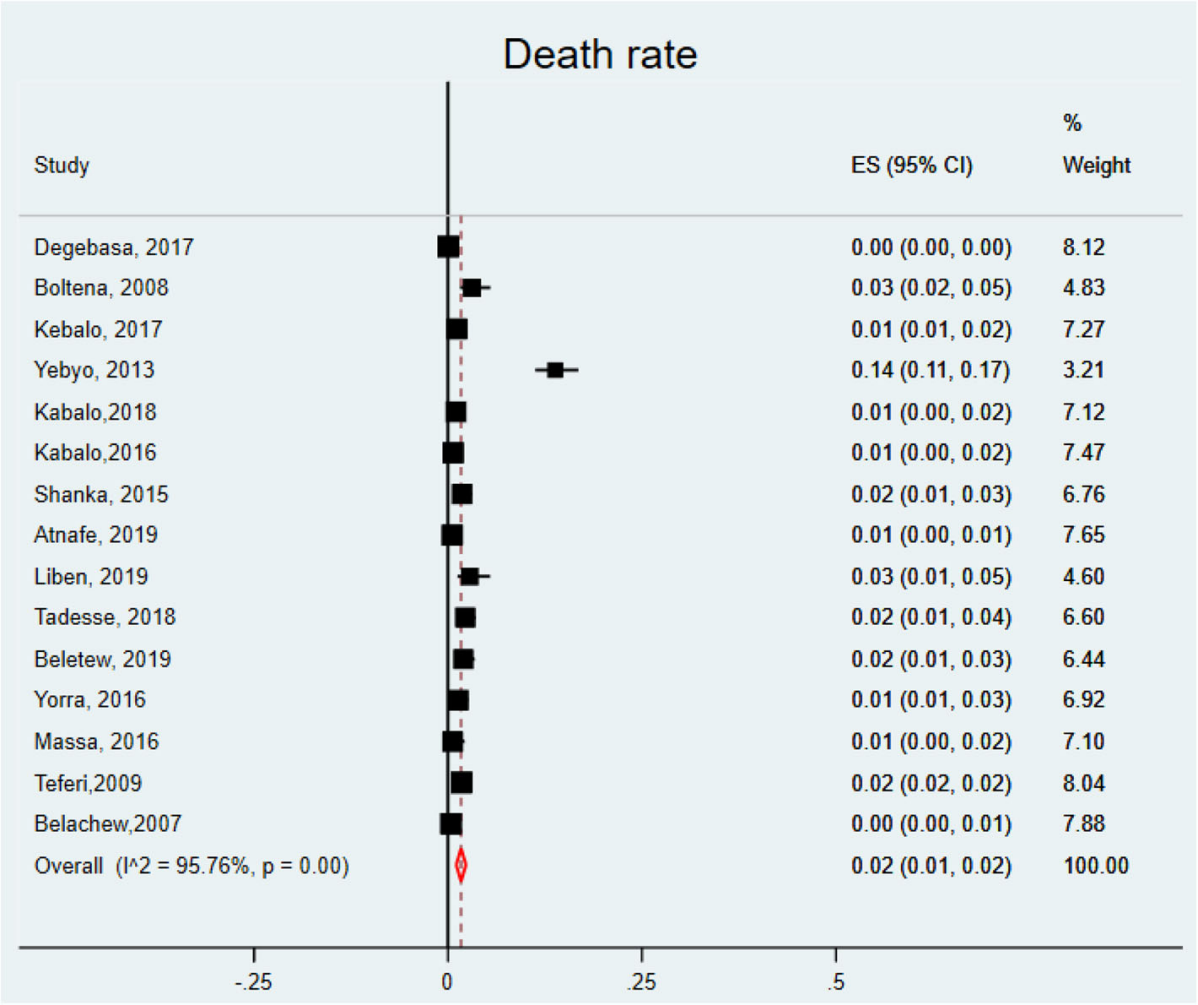
Forest plot showing the death rate of children with SAM treated in OTPs of Ethiopia, 2020

The other treatment outcomes were death rate, defaulter rate, and non-recovery rates. All were computed based on the random effect models due to the presence of significant heterogeneity. In this review, 15 studies [12, 18–20, 22–27, 31, 34, 35, 44, 45] were used to compute the pooled estimate of death rate. The minimum (0.05%) [20] and the maximum (13.85%) [25] death rates were reported from the studies conducted in Tigray region. In this meta-analysis, the pooled death rate was found to be 2% (95% CI: 1, 2, I^2^ = 95.76%, *P* = 0.000) (Fig. 4).

**Fig. 5:**
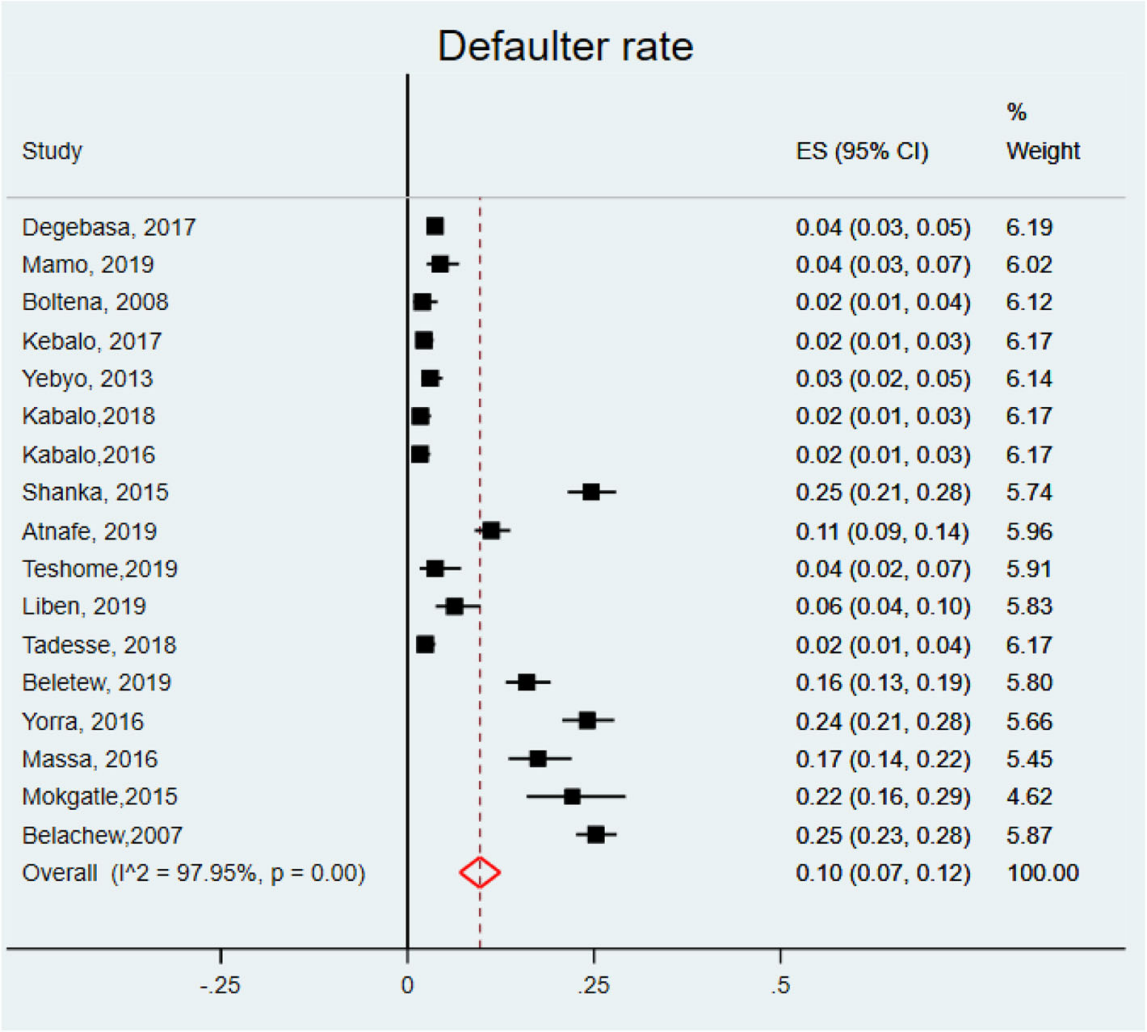
Forest plot showing the defaulter rate of children with SAM treated in OTPs of Ethiopia, 2020

**Fig. 6:**
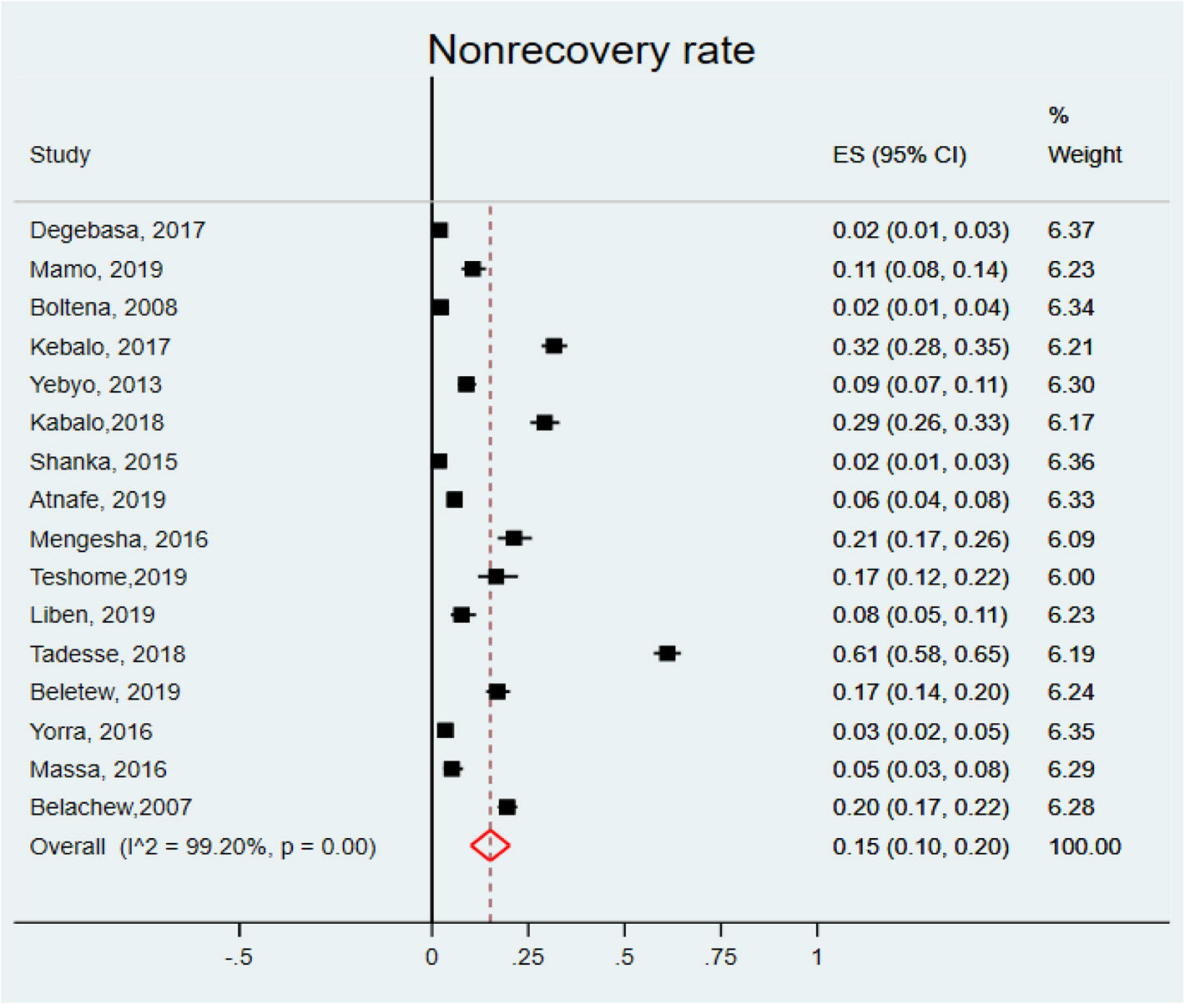
Forest plot showing the non-recovery rate of children with SAM treated in OTPs of Ethiopia, 2020

To compute the pooled estimates of defaulter rate of children with SAM from OTPs of Ethiopia, 17 studies [12, 18–28, 31, 33–35, 45] were used. The minimum defaulting rate (1.67%) was reported from a study conducted in SNNP [23] and the maximum one (25.2%) was from a study done in 2007 [12]. In our study, the defaulter rate was 10% (95%CI: 7, 12, I^2^ = 97.95%, *P* = 0.000) (Fig. 5). In the present study, the pooled estimate of non-recovery rate was computed from 16 studies [12, 18–22, 24–27, 31–35, 45]. From the included studies, a study conducted at the SNNP revealed that majority (61.13%) of children non-recovered and the lowest (1.83%) non-recovery rate was reported from a study done in the other part of SNNP. Non-recovery rate was 15% (95% CI: 10, 20, I^2^ = 99.2, *P* = 0.000) (Fig. 6).

### Subgroup analysis

As it is illustrated in Table 3, subgroup analysis was done using publication year, study region and study settings. This is done to explore the possible sources of heterogeneity of the included studies. Accordingly, six studies were done from 2007 to 2015 with the recovery rate 72% (95% CI: 62, 82) and the pooled recovery rate was lower in studies conducted after 2015 in Ethiopia (69, 95% CI: 62, 76). In accordance with the region where the studies were done, the highest recovery rate (72, 95% CI: 57, 87) was recorded form regions which were classified as others (Oromia, Afar, Dire Dawa, and (Oromia, Addis Ababa & SNNP)). The second higher recovery rate (71, 95% CI: 61, 80) was in SNNP which could be due to the fact that most the of studies were from this region. Lower recovery rates were recorded in Amhara and Tigray regions. In addition, the recovery rate was computed based on the study settings and children who were treated at health posts (66, 95% CI: 54, 77) had poor recovery rates as compared children who were treated in the health centers (71, 95% CI: 55, 86) (Table 3).

**Table 3:** Subgroup analysis of the recovery rate of under five children with SAM in the outpatient treatment programs of SAM in Ethiopia (*n* = 19)

Variables	Characteristics	Number of studies	Recovery rate (95% CI)
**Publication year**	< 2015	6	72% (62, 82)
	> 2015	13	69% (62, 76)
**Study Region**	Tigray	3	69% (62, 76)
	Amhara	2	65% (62, 68)
	SNNP	10	71% (61, 80)
	Others	4	72% (57, 87)
**Study setting**	HC	4	71% (55, 86)
	HP	8	66% (54, 77)
	HC & HP	7	75% (70, 80)

### Predictors of recovery rate of SAM children in Ethiopia

**Fig. 7:**
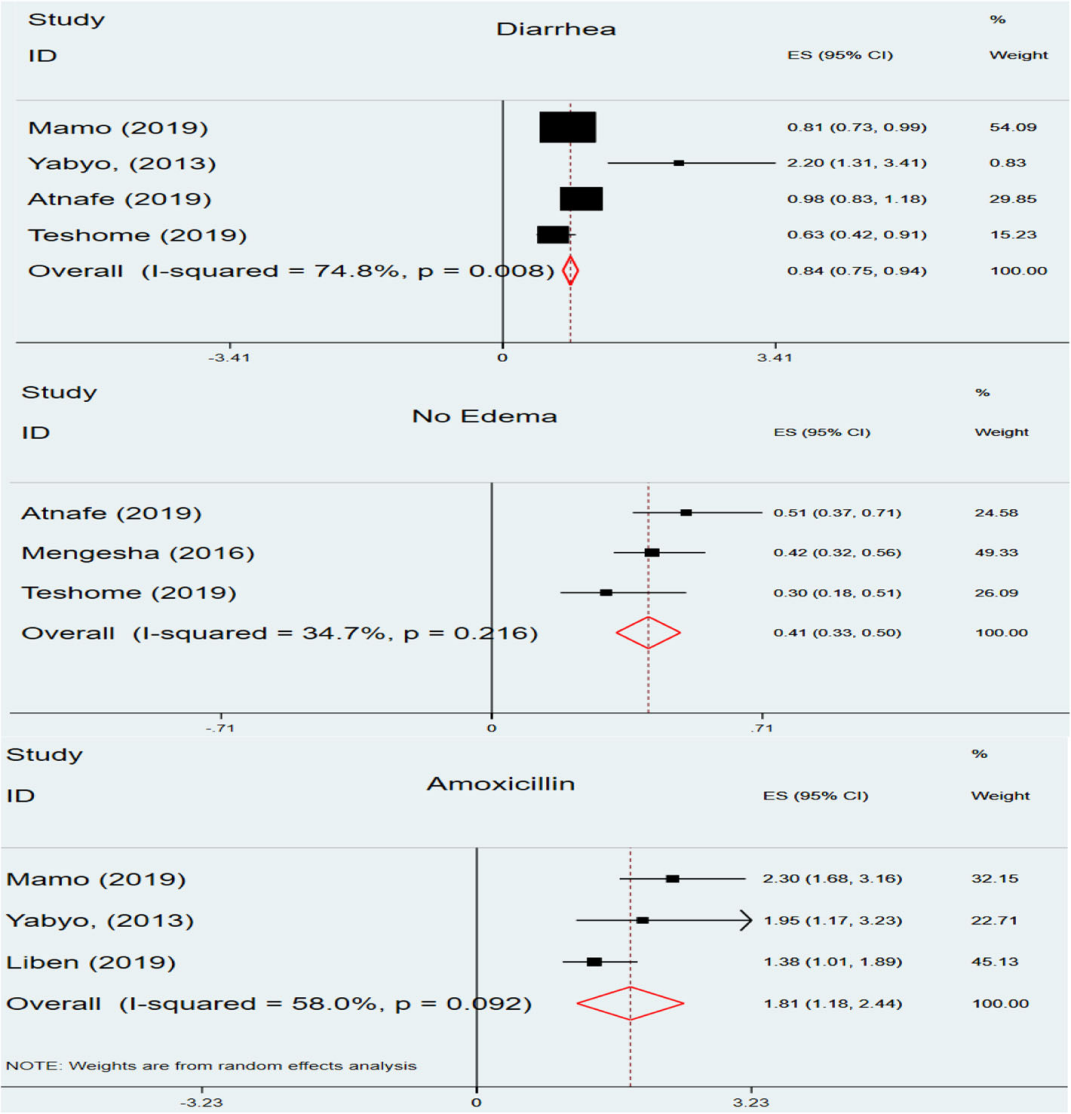
Forest plot showing predictors of recovery rate among under-five children with SAM in Ethiopia, 2020

In the current review, eight studies [21, 24–26, 31–34] presented the independent predictors of recovery rate using hazard ratios. The predictors which were reported by the original studies were diarrhea [21, 25, 31, 33], age > 24 months [26, 32, 33], no edema [31–33], deworming [21, 24, 25, 31, 34] and giving amoxicillin [21, 25, 34] as part of SAM process. The pooled estimates of hazard ratios revealed that age > 24 months (HR = 0.98, 95% CI: 0.81, 1.15, I^2^ = 80.8, *P* = 0.006) and deworming (HR = 1.04, 95% CI: 0.79, 1.28, I^2^ = 43.3, *P* = 0.133) were not significantly associated with recovery rate. However, diarrhea, no edema and Amoxicillin were found to be independent predictors of recovery rate (Fig. 7). The recovery rate of SAM children with diarrhea was 16% less likely compared to SAM children with no diarrhea (HR = 0.8, 95% CI: 0.75, 0.94). Similarly, the presence of no edema was found to be a prohibiting factor that decreased recovery rate by 41% (HR = 0.41, 95% CI: 0.33, 0.50). In addition, children who took amoxicillin were nearly two times more likely to recover early from SAM as compared to the counterparts (HR = 1.81, 95% CI:1.18, 2.44).

## Discussion

In this systematic review and meta-analysis, the treatment outcomes of under-five children with SAM admitted in outpatient therapeutic feeding programs of Ethiopia were determined. The treatment outcomes were recovery rate, death rate, defaulter rate and non-recovery rate. Besides, the predictors of recovery rate were analyzed using hazard ratios as an effect size estimator.

In the current study the proportion of recovery is found to be 70%, which is below the sphere standard, WHO and the national SAM management standards (recovery rate > 75%) [29, 46]. The possible elucidation for the low proportion of recovery could be associated with non-adherence of care givers of children to SAM treatment guidelines. Food sharing, trading of RUTFs as commodity, high cost of standard RUTFs, stigma associated with RUTFs use, lack of antibiotics, inappropriate exit from the program, and disliking the taste of RUTFs could also be the possible rationales for this lower recovery rate [7, 30]. This finding is comparable with the result of a systematic review about the recovery rate (70.5%) [47] of under five children in the inpatient therapeutic feeding programs in Ethiopia but lower than another review with the pooled recovery rate of 72.02% [48]. This could be due to high comorbidity rates in the inpatient therapeutic feeding programs as compared to the children in the OTPs. This finding is comparable with a study finding in Ghana with the recovery rate of 70.9% (346 out of 488) [49], but significantly higher than a study finding in Nigeria, where only 58% (4492 of 7742) of children get cured form SAM [50]. The current recovery rate is also lower than the findings of retrospective studies conducted in Cameroon and Pakistan, where 72.8% (185 of 254) & 89% (28,882 of 32,458) of children get recovered, respectively [51, 52]. The discrepancies could be attributed by differences in the number of study population, study design and the sociodemographic characteristics of the study participants as well as variation in the clinical expertise of health care providers.

In the present systematic review and meta-analysis, the pooled estimates of death rate (2%), defaulting rate (10%) and non-recovery rate (15%) were determined. The death rate is in line with the sphere and national standards [29, 46]. Likewise, the current finding is is in line with the death rate reported from Nigeria (2%) [50]. Nonetheless, this finding is higher than the study findings from Ghana (1.6%) [49], Cameroon (0.8%) [52] and Pakistan (0.4%) [51]. The possible explanations for the differences might be due to disparity in the organization of OTP centers, sociodemographic differences in study subjects and difference in the background of care takers of children. Regarding the defaulter rate, this finding coincides with what is recommended by the sphere standard, WHO and national SAM management standards (i.e. defaulting rate < 15%) [29, 46] . But the present result is below the findings of the study results in Pakistan [51], Ghana [49] and Nigeria [50] with defaulting rates of 10.6, 28.5 and 40%, respectively. All these findings are from the primary studies and this might be the possible reason for the variations. Differences in the therapeutic areas could also account for the discrepancies. This finding is also significantly lower than the original studies done in different parts of Ethiopia [12, 26–28, 35, 45], of which the proportion of the defaulting range from 16 to 25.2%. Moreover, the proportion of non-recovery (15%) is considerably higher in this meta-analysis. This is higher than most of the original studies included in this meta-analysis, but lower than the non-recovery rate from the study conducted in Cameroon (26.8%) [52]. This significant non-recovery rate could be attributed by high burden of comorbidities, inappropriate feeding process of the RUTFs, non-adherence to follow ups due to long distance to access RUTFs and drugs in some OTP centers of the country [7, 33–35]. Similarly, food insecurity could contribute to sharing food among family members and this may affect recovery rate of children [33].

Regarding the predictors of recovery rate, the presence of diarrhea, no edema and giving amoxicillin are independent predictors of recovery rate of children. The presence of diarrheal diseases as a comorbidity compromises recovery rate of under-five children by 16%. This is due to the fact that diarrhea and SAM are in vicious cycle. It delays the recovery rate of children with SAM as a result of metabolic disturbances, fluid and electrolyte losses and dehydration. These evidences are supported multitude of studies which implied diarrhea as a major determinant affecting the recovery rate of children with SAM [53–56]. Similarity, children with non-edematous SAM are found have lower recovery rates as compared to the counterparts edematous children. Those children having edema at admission are 59% more likely to recover within a short duration. This could be substantiated by the likelihood that children with edema might get better care by the health care providers and family members than wasted children [24, 57–59]. Moreover, giving amoxicillin for children with SAM as an empirical management is found to enhance survival of children. The likelihood of recovery of children who took amoxicillin is two times compared with children who didn’t take it. Severe acute malnutrition affects the whole system and it primarily compromises the immune system of children due to reductive adaption [60]. This paves the way for multiclausal infections which could delay the time of recovery of children [61]. Hence, empirical treatment of infections in the management process of SAM both in the inpatient and outpatient therapeutic feeding programs has a fundamental implication to improve survival of children [62]. Currently, the recommended antibiotic is amoxicillin and this is supported the systematic review and meta-analyses findings which were conducted prior to this systematic review [63, 64].

In general, the current study depicts that the treatment outcomes of children with SAM in OTP centers of Ethiopia is suboptimal. Specifically, the recovery and non-recovery rates are questionable. This could be due to incongruous implementation of the SAM treatment protocols that is corroborated by a systematic review in which treating SAM children in line with WHO recommendations improved childhood survival [65]. Besides, sharing RUTFs is the main challenge affecting the treatment outcomes of SAM children in Ethiopia [7, 30]. This could be because considerable proportions (0.9%) of children and adults have been living with HIV/AIDS that could increase RUTF consumers. Likewise, most of Ethiopian households are inhabited by extended families with the total fertility rate is 4.6 children per woman which perpetuates the food insecurities in the house hold level [66]. Hence, sharing RUTFs among family members could be inevitable.

### Strengths and limitations of the study

To our knowledge, this systematic review and meta-analysis is the first of its type in Ethiopia with so many strengths. The main strength of this systematic review and meta-analysis was that multiples reputable journals were explored comprehensively and exhaustively to retrieve all the original articles. All possible efforts are also made to communicate the primary authors to get articles that were difficult to access the full texts. The data were extracted using standardized and pretested extraction checklist. All possible analyses were done to estimate the pooled treatment outcomes and predictors of recovery rate of children with SAM in OTP centers of Ethiopia. These findings will also help policy makers, stakeholders, nongovernmental organizations and community health workers to modify their approach in the management process of children in OTPs. Despite these strengths, the current study has some limitations. Only articles that were published in the English language were included in this meta-analysis, which might affect the true estimates of treatment outcomes. To estimate the pooled predictors, limited numbers of studies were obtained and this might be the cause for under estimation predictors of recovery rates. In addition, the predictors were calculated using hazard ratios, but some studies reported factors using odds ratios. But none of the variables were found to have significant association with recovery. This could compromise the number of independent predictors.

## Conclusion

This meta-analysis revealed that the proportion of recovery and non-recovery were significantly higher than the sphere standard, WHO and national SAM management protocols. This finding is comparable with the recovery rate form inpatient units of the country which should not be really the case. This finding is alarming for policy makers and program implementers in Ethiopia. The presence of diarrheal disease as comorbidity and being non-edematous at admission were found to be prohibiting factors of time to recovery of children with SAM. In contrary, empirical treatment of children with amoxicillin was found to shorten the duration of recovery from SAM. Policy makers, community health workers, and program planners need to reconsider the community based management approaches of children with SAM.

## Supplementary information

**Additional file 1.** Critical Appraisal.

**Additional file 2.** PRISMA Checklist.

**Additional file 3.** Search String.

## Data Availability

All-important data for this study are included in the manuscript. If in need of additional data, they can be accessed from the corresponding author.

## Abbreviations

CI: Confidence interval
CMAM: Community based management of acute malnutrition
HR: Hazard ratio
OTP: Outpatient therapeutic feeding program
RUTF: Ready to use therapeutic food
SAM: Severe acute malnutrition
SNNP: Southern national, nationalities and peoples in Ethiopia
WHO: World health organization
