# Supplementary material for: Treatment outcomes of severe acute malnutrition and predictors of recovery in under-five children treated within outpatient therapeutic programs in Ethiopia: a systematic review and meta-analysis
Source: BMC Pediatr. 2020 Jul 7;20:335. doi: 10.1186/s12887-020-02188-5 (PMC7339430; doi:10.1186/s12887-020-02188-5)
Supplement: Supplementary file 1 — Additional file 1. Critical Appraisal. [file 12887_2020_2188_MOESM1_ESM.docx]

**The critical appraisal of cohort studies**

| Author, year | Q1 | | | | | Q2 | | | | Q3 | | | | Q4 | | | | Q5 | | | | Q6 | | | | Q7 | | | | Q8 | | | | Q9 | | | | Q10 | | | | Q11 | | | | Overall quality Result |
| --- | --- | --- | --- | --- | --- | --- | --- | --- | --- | --- | --- | --- | --- | --- | --- | --- | --- | --- | --- | --- | --- | --- | --- | --- | --- | --- | --- | --- | --- | --- | --- | --- | --- | --- | --- | --- | --- | --- | --- | --- | --- | --- | --- | --- | --- | --- |
|  | Y | | N | U | NA | Y | N | U | NA | Y | N | U | NA | Y | N | U | NA | Y | N | U | NA | Y | N | U | NA | Y | N | U | NA | Y | N | U | NA | Y | N | U | NA | Y | N | U | NA | Y | N | U | NA |  |
| Degebasa, 2017 | | √ |  |  |  | √ |  |  |  | √ |  |  |  |  |  | √ |  |  |  | √ |  | √ |  |  |  | √ |  |  |  | √ |  |  |  | √ |  |  |  |  |  | √ |  |  | √ |  |  | 7/11 (63.64%) |
| Mamo, 2019 | | √ |  |  |  | √ |  |  |  | √ |  |  |  | √ |  |  |  | √ |  |  |  | √ |  |  |  | √ |  |  |  | √ |  |  |  |  |  | √ |  | √ |  |  |  | √ |  |  |  | 10/11 (90.9%) |
| Yabyo, 2013 | | √ |  |  |  | √ |  |  |  | √ |  |  |  | √ |  |  |  | √ |  |  |  | √ |  |  |  | √ |  |  |  | √ |  |  |  |  |  | √ |  | √ |  |  |  | √ |  |  |  | 10/11 (90.9%) |
| Kabalo,2018 | | √ |  |  |  | √ |  |  |  | √ |  |  |  | √ |  |  |  | √ |  |  |  | √ |  |  |  | √ |  |  |  | √ |  |  |  |  |  | √ |  |  |  | √ |  | √ |  |  |  | 9/11 (81.82%) |
| Shanka, 2015 | | √ |  |  |  | √ |  |  |  | √ |  |  |  | √ |  |  |  | √ |  |  |  | √ |  |  |  | √ |  |  |  | √ |  |  |  |  |  | √ |  | √ |  |  |  | √ |  |  |  | 10/11 (90.9%) |
| Atnafe, 2019 | | √ |  |  |  | √ |  |  |  | √ |  |  |  | √ |  |  |  | √ |  |  |  | √ |  |  |  | √ |  |  |  | √ |  |  |  |  |  | √ |  | √ |  |  |  | √ |  |  |  | 10/11 (90.9%) |
| Mengesha,  2016 | | √ |  |  |  | √ |  |  |  | √ |  |  |  | √ |  |  |  | √ |  |  |  | √ |  |  |  | √ |  |  |  | √ |  |  |  |  |  | √ |  |  |  | √ |  | √ |  |  |  | 9/11 (81.82%) |
| Teshome,  2019 | | √ |  |  |  | √ |  |  |  | √ |  |  |  | √ |  |  |  | √ |  |  |  | √ |  |  |  | √ |  |  |  | √ |  |  |  |  |  | √ |  |  |  | √ |  | √ |  |  |  | 9/11 (81.82%) |
| Liben, 2019 | | √ |  |  |  | √ |  |  |  | √ |  |  |  | √ |  |  |  | √ |  |  |  | √ |  |  |  | √ |  |  |  | √ |  |  |  |  |  | √ |  | √ |  |  |  | √ |  |  |  | 10/11 (90.9%) |
| Tadesse, 2018 | | √ |  |  |  | √ |  |  |  | √ |  |  |  |  |  | √ |  |  |  | √ |  | √ |  |  |  | √ |  |  |  | √ |  |  |  |  |  | √ |  |  |  | √ |  |  | √ |  |  | 6/11 (54.54%) |
| Beletew, 2019 | | √ |  |  |  | √ |  |  |  | √ |  |  |  | √ |  |  |  | √ |  |  |  | √ |  |  |  | √ |  |  |  | √ |  |  |  |  |  | √ |  |  |  | √ |  |  | √ |  |  | 8/11 (72.72%) |
| Yorra, 2016 | | √ |  |  |  | √ |  |  |  | √ |  |  |  |  |  | √ |  |  |  | √ |  | √ |  |  |  | √ |  |  |  | √ |  |  |  |  |  | √ |  |  |  | √ |  | √ |  |  |  | 7/11 (63.64%) |
| Massa, 2016 | | √ |  |  |  | √ |  |  |  | √ |  |  |  | √ |  |  |  | √ |  |  |  | √ |  |  |  | √ |  |  |  | √ |  |  |  |  |  | √ |  | √ |  |  |  | √ |  |  |  | 10/11 (90.9%) |
| Teferi,2009 | | √ |  |  |  | √ |  |  |  | √ |  |  |  | √ |  |  |  | √ |  |  |  | √ |  |  |  | √ |  |  |  | √ |  |  |  |  |  | √ |  |  |  | √ |  |  | √ |  |  | 8/11 (72.72%) |

****Y=yes, N=no, U=unclear, NA=not applicable, <60%=low, 60-80%=medium, >80%=high quality***

**Critical appraisal of cross sectional studies**

| Author, year | Q1 | | | | Q2 | | | | Q3 | | | | | Q4 | | | | | Q5 | | | | Q6 | | | | Q7 | | | | Q8 | | | | Overall quality result |
| --- | --- | --- | --- | --- | --- | --- | --- | --- | --- | --- | --- | --- | --- | --- | --- | --- | --- | --- | --- | --- | --- | --- | --- | --- | --- | --- | --- | --- | --- | --- | --- | --- | --- | --- | --- |
|  | Y | N | U | NA | Y | N | U | NA | | Y | N | U | NA | | Y | N | U | NA | Y | N | U | NA | Y | N | U | NA | Y | N | U | NA | Y | N | U | NA |  |
| Boltena, 2008 | √ |  |  |  | √ |  |  |  | | √ |  |  |  | | √ |  |  |  |  |  |  | √ |  |  |  | √ | √ |  |  |  | √ |  |  |  | 6/8(75%) |
| Kabalo, 2017 | √ |  |  |  | √ |  |  |  | | √ |  |  |  | | √ |  |  |  | √ |  |  |  | √ |  |  |  | √ |  |  |  | √ |  |  |  | 8/8(100%) |
| Kabalo,2016 | √ |  |  |  | √ |  |  |  | | √ |  |  |  | | √ |  |  |  | √ |  |  |  | √ |  |  |  | √ |  |  |  | √ |  |  |  | 8/8(100%) |
| Mokgatle,2015 | √ |  |  |  | √ |  |  |  | | √ |  |  |  | | √ |  |  |  |  |  |  | √ |  |  |  | √ | √ |  |  |  | √ |  |  |  | 6/8(75%) |
| Belachew, 2007 | √ |  |  |  | √ |  |  |  | | √ |  |  |  | | √ |  |  |  |  |  |  | √ |  |  |  | √ | √ |  |  |  | √ |  |  |  | 6/8(75%) |

****Y=yes, N=no, U=unclear, NA=not applicable, <60%=low, 60-80%=medium, >80%=high quality***
