# Supplementary material for: Treatment outcomes of severe acute malnutrition and predictors of recovery in under-five children treated within outpatient therapeutic programs in Ethiopia: a systematic review and meta-analysis
Source: BMC Pediatr. 2020 Jul 7;20:335. doi: 10.1186/s12887-020-02188-5 (PMC7339430; doi:10.1186/s12887-020-02188-5)
Supplement: Supplementary file 3 — Additional file 3. Search String. [file 12887_2020_2188_MOESM3_ESM.docx]

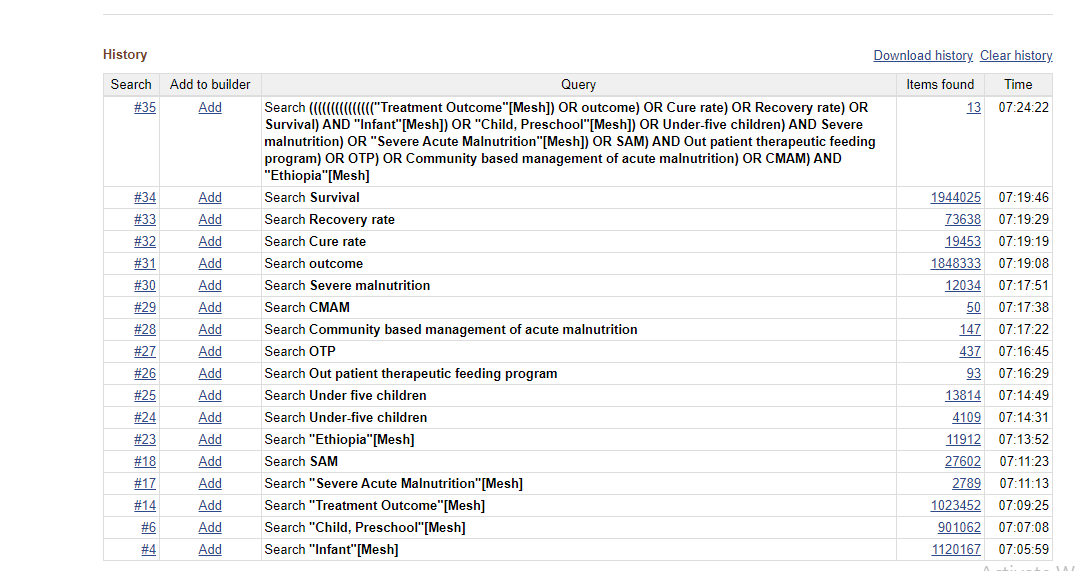


Pubmed


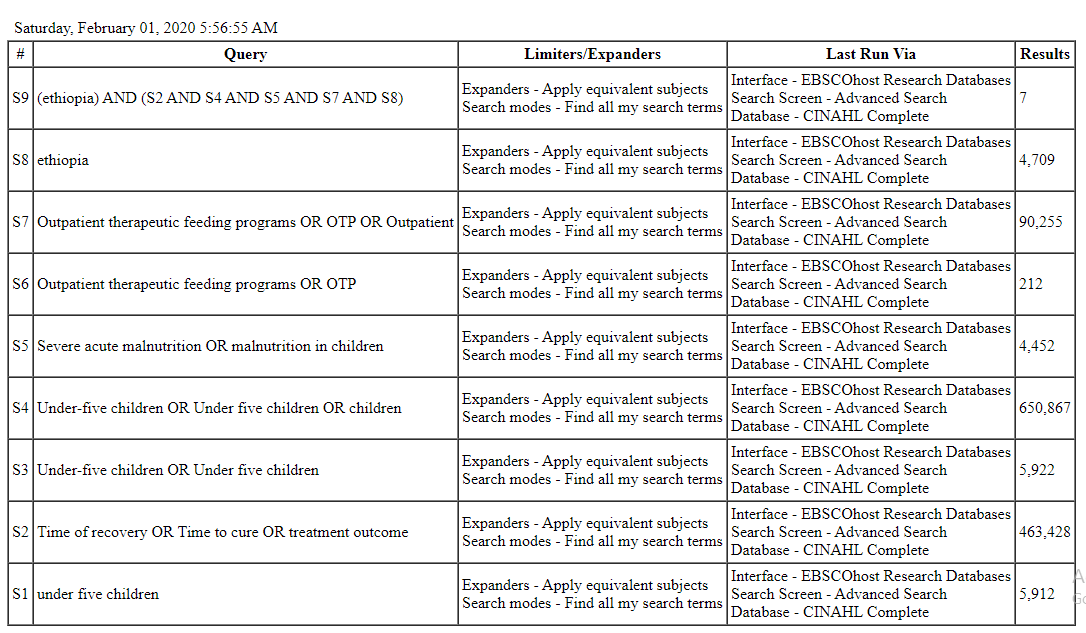


CINAHL


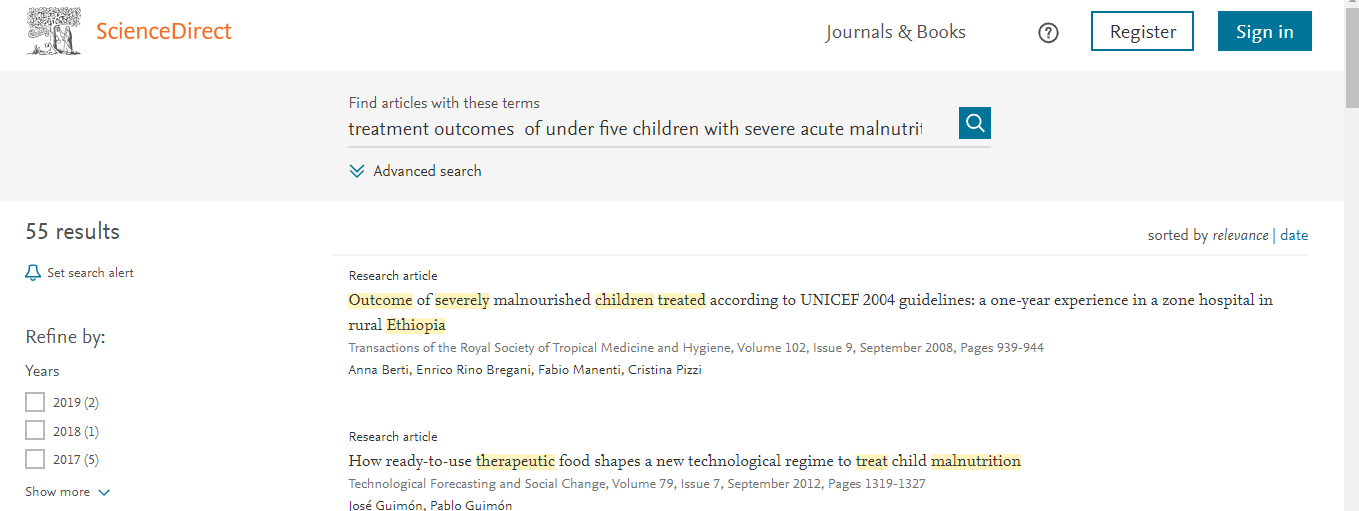


Sceince Direct


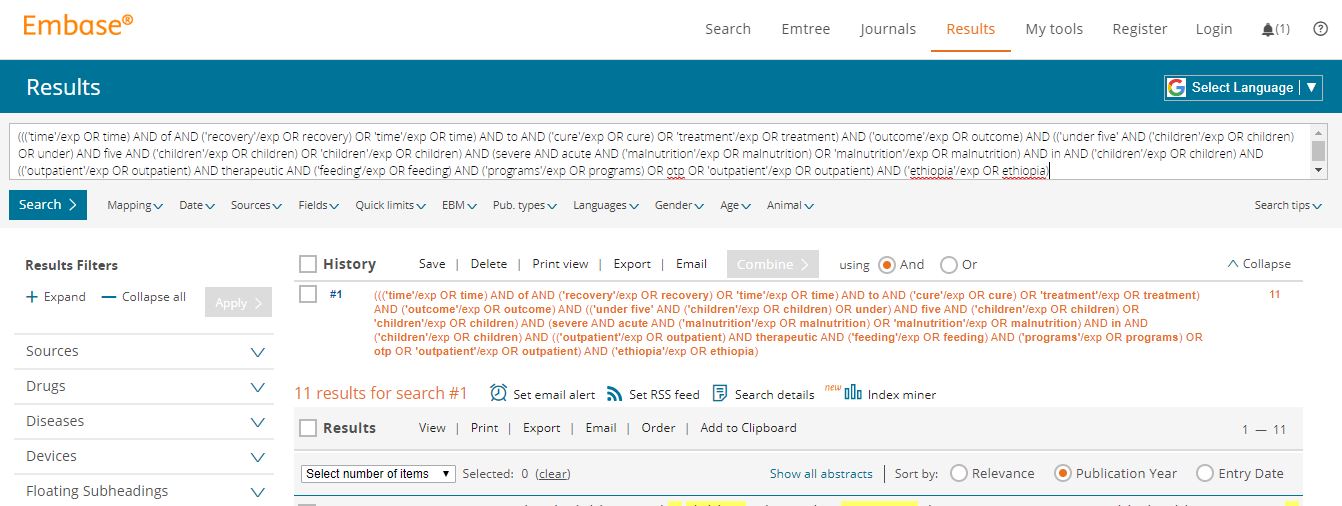


Embase


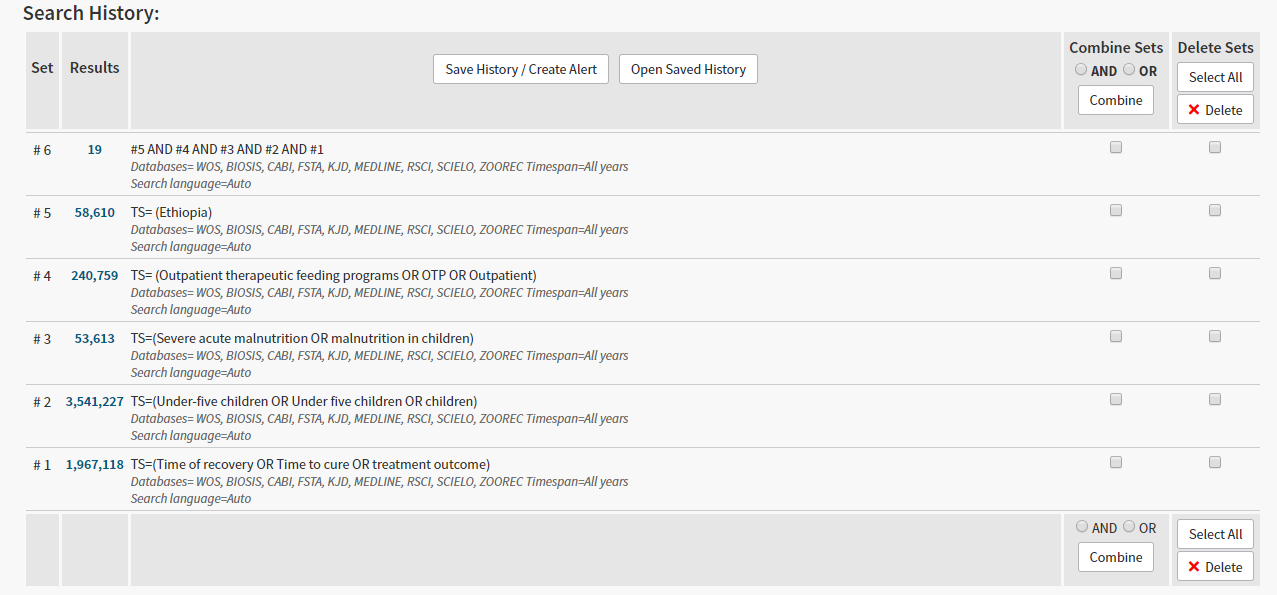


Web science

Top of Form

[**Accessibility Information and Tips**](javascript:openWideTip('http://support.ebsco.com.ludwig.lub.lu.se/help/?int=ehost&lang=en&feature_id=access&TOC_ID=Always&SI=0&BU=0&GU=1&PS=0&ver=&dbs=cmedm'))Revised Date: 07/2015

**Print Search History**

| 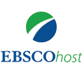 | Saturday, February 01, 2020 6:41:49 AM |
| --- | --- |

| **#** | **Query** | **Limiters/Expanders** | **Last Run Via** | **Results** |
| --- | --- | --- | --- | --- |
| S5 | S1 AND S2 AND S3 AND S4 | Expanders - Apply equivalent subjects Search modes - Find all my search terms | Interface - EBSCOhost Research Databases Search Screen - Advanced Search Database - MEDLINE | 15 |
| S4 | Ethiopia | Expanders - Apply equivalent subjects Search modes - Find all my search terms | Interface - EBSCOhost Research Databases Search Screen - Advanced Search Database - MEDLINE | 19,619 |
| S3 | Outpatient therapeutic feeding programs OR OTP OR Outpatient | Expanders - Apply equivalent subjects Search modes - Find all my search terms | Interface - EBSCOhost Research Databases Search Screen - Advanced Search Database - MEDLINE | 183,790 |
| S2 | Under-five children OR Under five children OR children | Expanders - Apply equivalent subjects Search modes - Find all my search terms | Interface - EBSCOhost Research Databases Search Screen - Advanced Search Database - MEDLINE | 1,339,001 |
| S1 | Time of recovery OR Time to cure OR treatment outcome | Expanders - Apply equivalent subjects Search modes - Find all my search terms | Interface - EBSCOhost Research Databases Search Screen - Advanced Search Database - MEDLINE | 1,522,472 |

Bottom of Form

Medline
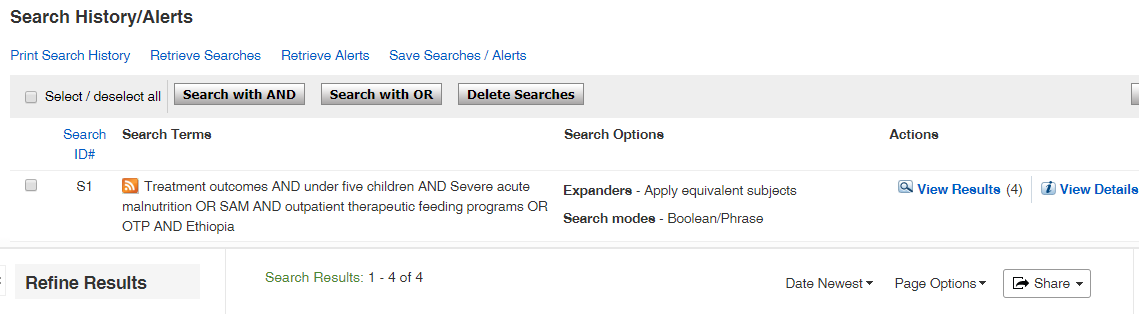


FSTA


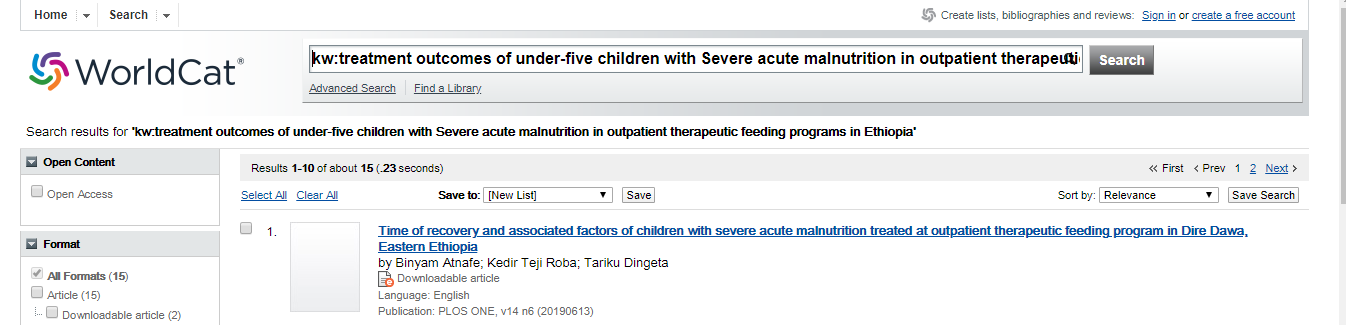


World Cat


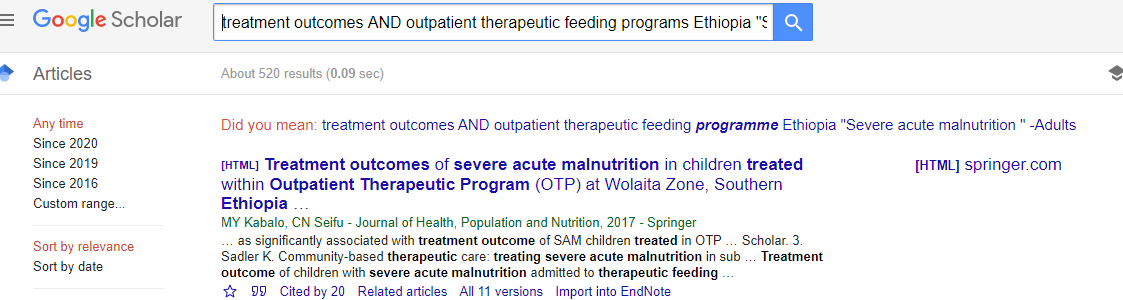


Google scholar
